# Construction and validation of a prognostic nomogram for anal squamous cell carcinoma

**DOI:** 10.1002/cam4.4458

**Published:** 2021-12-01

**Authors:** Ningning Yang, Lu Xu, Qingqing Wang, Fengxia Chen, Yunfeng Zhou

**Affiliations:** ^1^ Department of Radiation Oncology and Medical Oncology Zhongnan Hospital of Wuhan University Wuhan China; ^2^ Hubei Cancer Clinical Study Center, Hubei Key Laboratory of Tumor Biological Behaviors Zhongnan Hospital of Wuhan University Wuhan China; ^3^ Department of Ultrasound Zhongnan Hospital of Wuhan University Wuhan China

**Keywords:** anus squamous cell carcinoma, nomogram, prognosis, risk stratification, SEER

## Abstract

**Background:**

Anal squamous cell carcinoma (ASCC) is the main subtype of anal cancer and has great heterogeneity in prognosis. We aimed to construct a nomogram for predicting their 1‐, 3‐, and 5‐year overall survival (OS) rates.

**Methods:**

Patients with ASCC, enrolled between January 1, 2010 and December 31, 2017, were identified from the SEER database. They were divided into a training group and a validation group in a ratio of 7:3. Univariate and multivariate Cox analyses were used to identify the prognostic factors for OS. Then a prognostic nomogram was established and validated by Harrell consistency index (C‐index), area under the curve (AUC) of the receiver operating characteristic (ROC) curves, calibration plots, and decision curve analysis (DCA).

**Results:**

We identified 761 patients in training group and 326 patients in validation group. Four prognostic factors including age, sex, AJCC stage, and radiotherapy were identified and integrated to construct a prognostic nomogram. The C‐index and AUC values proved the model's effectiveness and calibration plots manifested its excellent discrimination. Furthermore, in comparison to the AJCC stage, the C‐index, AUC, and DCA proved the nomogram to be of good predictive value. Finally, we constructed a risk stratification model for dividing patients into low‐risk, medium‐risk, and high‐risk groups, and there were obvious differences in OS.

**Conclusions:**

A prognostic nomogram was firstly established for predicting the survival probability of ASCC patients and helping clinicians improve their risk management.

## Graphical abstract

First, from the SEER database, we identified the prognostic factors for anal squamous cell carcinoma (ASCC) patients and develop a nomogram to better predict the prognosis of ASCC patients. Secondly, through comparison, our predictive model is a further improvement of traditional AJCC staging system based on patient’s demographic and clinicopathological features not only in survival prediction, but also in clinical utility.

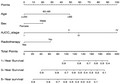

## 1 INTRODUCTION

Anal squamous cell carcinoma (ASCC), a rare malignant cancer but very common subtype of anal cancer, accounted for less than 0.3% of all new cancer cases in 2020.^1^ The incidence of ASCC is low but has been increasing, particularly in elderly patients.^2^, ^3^, ^4^ The most common symptoms were the appearance of a mass, anal pain, bleeding, local ulcers, or fecal incontinence; however, some patients had no clinical symptoms.^5^ Eighty percent to 90% of patients are diagnosed with locoregional disease and could receive definitive treatments to achieve good survival outcomes,^6^ with a 3‐year progression‐free survival (PFS) rates ranging from 60% to 80%^7^; meanwhile 10% of patients present with distant metastasis at initial diagnosis, having a particularly poor prognosis with a 5‐year survival rate of approximately 30%.^8^

Patients with ASCC have great heterogeneity in clinical manifestations and therapeutic efficacy, which leads to different survival outcomes. It is erroneous to merely depend on the AJCC staging system to predict the prognosis of cancer patients. Currently, the AJCC staging system does not incorporate demographic and clinicopathological characteristics such as age, sex, and treatment strategy, but literature has shown that they are highly correlated with the prognosis of ASCC patients.^2^, ^9^, ^10^, ^11^, ^12^ Researchers have reported that some hematological and immunohistochemical indicators may play a prognostic role in patients with ASCC who received definitive treatment.^13^, ^14^, ^15^ Casadei‐Gardini et al. enrolled 308 ASCC patients to develop a logistic nomogram using the pre‐treatment systemic inflammation index (platelet × neutrophil/lymphocyte), pre‐treatment nodal status and pre‐treatment hemoglobin levels in order to predict the prognosis of patients treated with concurrent chemoradiation.^16^ However, for patients newly diagnosed with ASCC, there is a lack of effective prognostic models to better predict individual patient survival outcomes. Clinicians are more likely to have access to clinicopathological information for cancer patients. Therefore, the clinical need for risk assessment and recommendations for treatment impelled us to develop a clinicopathological information‐based model to predict the survival of patients with ASCC.

The nomogram is a practical and effective tool to predict individual risk and has exhibited an important role in patient survival prediction, risk evaluation, and clinical decision in oncology.^17^, ^18^, ^19^ Analysts usually take significant predictors and integrate them into a graphic to predict a patient's overall survival (OS) rate. In addition, the nomogram usually exerts a superiority in predicting individual risk than the AJCC temporary storage system.

Here, we used the SEER database to identify the clinical and pathological characteristics of ASCC and develop a prognostic nomogram, in order to provide a more accurate survival prediction for ASCC patients and help clinicians evaluate prognostic risk and make the optimal treatment decisions.

## 2 MATERIALS AND METHODS

### 2.1 Data source and patient selection

The SEER Program contains information on cancer statistics of approximately 28% of the US population. Patient’ data were downloaded from SEER*Stat version 8.3.8 (accession number: 10736‐Nov 2019). The SEER database is open access; hence, there is no need for an ethics committee review approval.

From the SEER database, the location codes collected included malignant neoplasms C21.0 (Anus, NOS), C21.1 (anal canal), C21.2 (Cloacogenic region), and C21.8 (overlapping lesions of the rectum, anus, and anal canal). Subsequently, we filtered the patients on the basis of ICD‐O‐3 histology/behavior codes, including verrucous carcinoma, NOS (8051/3), squamous cell carcinoma, NOS (8070/3), squamous cell carcinoma, keratinization, NOS (8071/3), squamous cell carcinoma, large cell, non‐keratinization, NOS (8072/3), squamous cell carcinoma, small cell, non‐keratinization (8073/3), squamous cell carcinoma, micro‐invasive (8076/3), and basaloid squamous cell carcinoma (8083/3).

**FIGURE 1:**
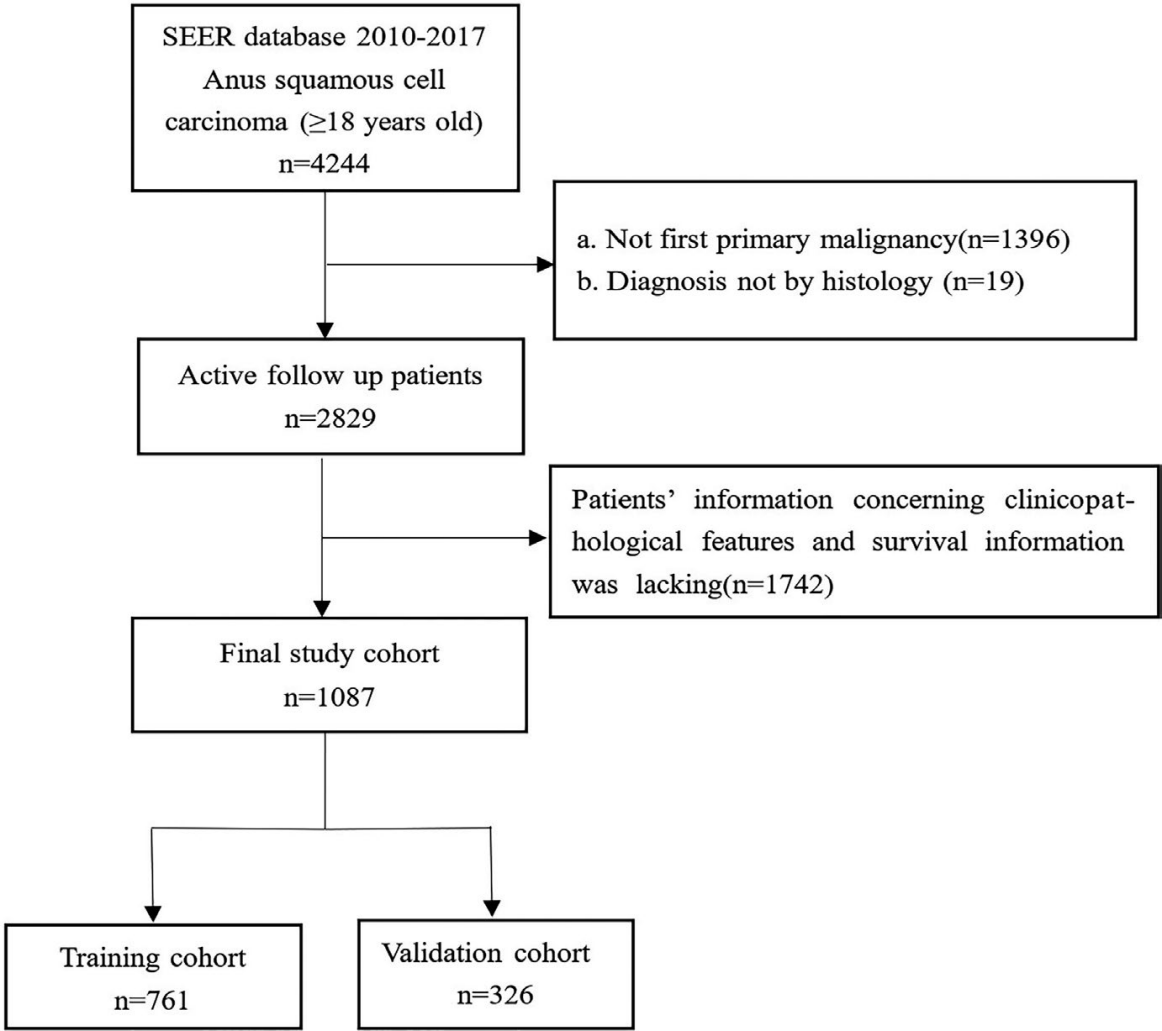
Patient selection flowchart

Patients enrolled in our study fulfilled the following inclusion criteria: (a) patients with ASCC were enrolled from January 1, 2010 to December 31, 2017, (b) ASCC was their only primary malignancy, (c) patients were diagnosed with histological methods, and (d) patients’ follow‐up data including survival time and vital status were complete. Patients were excluded under the following conditions: (a) being under 18 years of age. (b) incomplete demographic or clinicopathological information, including age, sex, race, tumor grade, tumor size, AJCC stage, T stage, N stage, or M stage, and regardless of the order of the treatment, uncertain treatment information (whether they underwent surgery, radiotherapy, or chemotherapy), and (c) diagnosis by death certificate or autopsy. In addition, the AJCC stage was redefined according to the 2017 version. The detailed screening procedure is shown in Figure 1.

The following factors were selected and sorted: age at diagnosis, gender, marital status, race, tumor size, tumor differentiation, AJCC stage, N stage, M stage, therapy, survival months, and survival status. For tumor size, the optimal cutoff point was calculated using the X‐tile software (Figure S1). OS was the main endpoint. OS refers to the time from diagnosis to death or the last follow‐up.

### 2.2 Construction and validation of the nomogram

In this study, 1087 patients with ASCC were included. Through the “caret” package, we indiscriminately divided patients into two cohorts in the ratio of 7:3. The training cohort (*n* = 761) was applied to develop and internally validate nomograms, while the validation group (*n* = 326) was used for external validation.

In the training cohort, univariable and multivariable Cox proportional hazard regression models were used to identify significant predictors of OS, then, the nomogram was constructed to estimate ASCC patients’ 1‐, 3‐, and 5‐year survival rate according to the proportional conversion of each regression coefficient into a 0–100 point scale through the “rms” package. Furthermore, we identified the reliability of the model through internal and external validation. C‐index, the area under the curve (AUC) of the receiver operating characteristic (ROC) curves, and calibration curves were employed to evaluate the discrimination of the model. The C‐index is between 0 and 1.0, of which 1.0 represents the best prediction and 0.5 stands for a completely random predictor. The AUC value ranges from 0.5 to 1, and there is a positive correlation between the value and predictive ability. Calibration maps were employed to test the difference between the predicted and observed survival rates. Decision curve analysis (DCA) was conducted to identify the clinical application of the model. Based on the total score of every patient according to the prognostic nomogram, patients were classified into low‐, intermediate‐, and high‐risk groups by X‐tile software.

### 2.3 Statistical analyses

Summary statistics were used to depict the basic characteristics of the included population. The Chi‐square test was used to compare categorical differences between different groups. Univariate and multivariate Cox regression analyses were used to analyze the prognostic factors associated with OS. Kaplan–Meier curves and log‐rank tests were used to assess survival differences. Moreover, a prognostic nomogram model was developed to predict the survival probabilities of patients with ASCC at 1‐, 3‐, and 5‐years. The values of C‐index and AUC were used to evaluate the model's discrimination. Calibration maps were applied to assess the difference between the observed and predicted probabilities of survival, and the DCA was used to assess its clinical utility. Analysis was performed using X‐tile software, SPSS (version 24.0; SPSS, Inc.), GraphPad Prism (version 8.0; GraphPad), and packages (rms, survival, survival ROC, hmisc, rmda, etc.) in R software version 3.6.6 (http://www.rproject.org). Statistical testing was bilateral and significance was set at *p *< 0.05.

## 3 RESULTS

### 3.1 Patient characteristics

We identified 1087 ASCC cases from the SEER database, including 761 patients in the training group and 326 patients in the validation group (Figure 1). The patients’ demographic and clinical features are exhibited in Table 1. No significant differences existed in demographic and clinicopathological distribution between the training and validation groups. For all the patients with ASCC, the mean and median ages were 60.9 and 60 years, respectively. Nearly half of the patients were between 60 and 65 years of age. A total of 685 (63%) patients were female and 654 (60.2%) were single. Most of the patients (915, 84.2%) were White. Most of the ASCC patients presented with grade II/III (84.2%), AJCC stage II/III (72.3%), and a tumor size smaller than 3.5 cm (55.7%). In terms of AJCC stage, a few patients were classified as T2 (49.2%), N0 (65.2%), and M0 (94%). For treatment, more than four‐fifths of the patients had undergone radiotherapy (87.4%) and chemotherapy (85.9%), with less than half of the patients receiving surgery (40.8%). Of these patients, 273 (25.1%) died and the 1‐, 3‐, and 5‐year OS rates were 74.8%, 41.2%, and 20.5%, respectively.

**TABLE 1:** Basic demographic and clinicopathological features of ASCC patients

Variables	All patients *n* (%)	Training cohort *n* (%)	Validation cohort *n* (%)	*p* value
Total	1087 (100)	761 (100)	326 (100)	
Age, years				0.782
≤50	197 (18.1)	142 (18.7)	55 (16.9)	
50–65	549 (50.5)	382 (50.2)	167 (51.2)	
>65	341 (31.4)	237 (31.1)	104 (31.9)	
Sex				0.308
Male	402 (37)	274 (36)	128 (39.3)	
Female	685 (63)	487 (64)	198 (60.7)	
Marital status				0.907
Married	433 (39.8)	304 (39.9)	129 (39.6)	
Single	654 (60.2)	457 (60.1)	197 (60.4)	
Race				0.895
White	915 (84.2)	638 (83.8)	277 (85)	
Black	133 (12.2)	95 (12.5)	38 (11.7)	
Others	39 (3.6)	28 (3.7)	11 (3.4)	
Grade				0.388
1	164 (15.1)	124 (16.3)	40 (12.3)	
2	516 (47.5)	356 (46.8)	160 (49.1)	
3	399 (36.7)	275 (36.1)	124 (38)	
4	8 (0.7)	6 (0.8)	2 (0.6)	
Tumor size, cm				0.558
≤3.5	606 (55.7)	418 (54.9)	188 (57.7)	
3.5–6	334 (30.7)	235 (30.9)	99 (30.4)	
≥6	147 (13.5)	108 (14.2)	39 (12)	
AJCC stage				0.939
I	237 (21.8)	163 (21.4)	74 (22.7)	
II	431 (39.7)	301 (39.6)	130 (39.9)	
III	354 (32.6)	250 (32.9)	104 (31.9)	
IV	65 (6)	47 (6.2)	18 (5.5)	
AJCC T				0.115
T1	278 (25.6)	194 (25.5)	84 (25.8)	
T2	535 (49.2)	372 (48.9)	163 (50)	
T3	179 (16.5)	136 (17.9)	43 (13.2)	
T4	95 (8.7)	59 (7.8)	36 (11)	
AJCC N				0.456
N0	709 (65.2)	491 (64.5)	218 (66.9)	
N1	378 (34.8)	270 (35.5)	108 (33.1)	
AJCC M				0.677
M0	1022 (94)	714 (93.8)	308 (94.5)	
M1	65 (6)	47 (6.2)	18 (5.5)	
Surgery				0.672
No	644 (59.2)	454 (59.7)	190 (58.3)	
Yes	443 (40.8)	307 (40.3)	136 (41.7)	
Radiotherapy				0.415
No	137 (12.6)	100 (13.1)	37 (11.3)	
Yes	950 (87.4)	661 (86.9)	289 (88.7)	
Chemotherapy				0.866
No	153 (14.1)	108 (14.2)	45 (13.8)	
Yes	934 (85.9)	653 (85.8)	281 (86.2)	
1‐year OS (%)	74.8	74.4	75.2	‐
3‐year OS (%)	41.2	41.7	40.2	‐
5‐year OS (%)	20.5	20.5	20.6	‐

Abbreviations: OS, overall survival.

### 3.2 Independent predictors in the training group

In the training group, the prognostic considerations of OS were identified using univariate and multivariate Cox proportional hazard regression analyses (Table 2). Results from univariate analyses showed that age, sex, marital status, race, tumor size, AJCC stage, T, N, M stage, surgical operation, and radiotherapy were significant independent risk factors for patients with ASCC (*p *< 0.05).

**TABLE 2:** Univariate and multivariate Cox proportional hazards analyses for OS in training cohort of ASCC patients

Variables	Univariate analysis		Multivariate analysis	
	HR (95% CI)	*p* value	HR (95% CI)	*p* value
Age, years				
≤50	Reference		Reference	
50–65	1.487 (0.934–2.343)	0.087	1.886 (1.165–3.052)	**0.010**
>65	2.546 (1.617–4.011)	**<0.001**	2.983 (1.839–4.840)	**<0.001**
Sex				
Male	Reference		Reference	
Female	0.739 (0.555–0.984)	**0.038**	0.642 (0.473–0.870)	**0.004**
Marital status				
Married	Reference		Reference	
Single	1.532 (1.125–2.085)	**0.007**	1.253 (0.908–1.728)	0.170
Race				
White	Reference		Reference	
Black	1.339 (0.900–1.993)	0.15	1.371 (0.895–2.099)	0.147
Others	1.807 (1.003–3.255)	**0.049**	1.591 (0.873–2.901)	0.129
Tumor size, cm				
≤3.5	Reference		Reference	
3.5–6	1.682 (1.215–2.329)	**0.002**	1.355 (0.901–2.039)	0.144
≥6	3.493 (2.411–5.059)	**<0.001**	1.827 (0.975–3.422)	0.060
Grade				
1	Reference		—	
2	1.214 (0.790–1.867)	0.376	—	
3	1.179 (0.758–1.835)	0.465	—	
4	1.614 (0.384–6.791)	0.514	—	
AJCC stage				
I	Reference		Reference	
II	2.367 (1.447–3.872)	**0.001**	3.788 (1.614–8.891)	**0.002**
III	2.759 (1.677–4.537)	**<0.001**	2.046 (0.661–6.335)	0.214
IV	6.940 (3.825–12.590)	**<0.001**	4.786 (1.505–15.226)	**0.008**
AJCC T				
T1	Reference		Reference	
T2	1.691 (1.118–2.558)	**0.013**	0.533 (0.254–1.120)	0.097
T3	3.415 (2.177–5.358)	**<0.001**	0.717 (0.300–1.716)	0.455
T4	3.078 (1.745–5.427)	**<0.001**	0.985 (0.397–2.445)	0.975
AJCC N				
N0	Reference		Reference	
N1	1.760 (1.323–2.341)	**<0.001**	1.622 (0.697–3.776)	0.262
AJCC M				
M0	Reference		—	
M1	3.223 (2.097–4.953)	**<0.001**	—	—^a^
Surgery				
No	Reference		Reference	
Yes	0.661 (0.490–0.892)	**0.007**	0.807 (0.566–1.150)	0.236
Radiotherapy				
No	Reference		Reference	
Yes	0.655 (0.452–0.949)	**0.025**	0.559 (0.365–0.857)	**0.008**
Chemotherapy				
No	Reference		—	
Yes	0.766 (0.529–1.111)	0.16	—	

Abbreviations: CI, confidence interval; HR, hazard ratio.

Bold values indicate *p* < 0.05 in univariate and multivariate analyses.

^a^ Linearly dependent covariates AJCC M = IV of AJCC stage.

**FIGURE 2:**
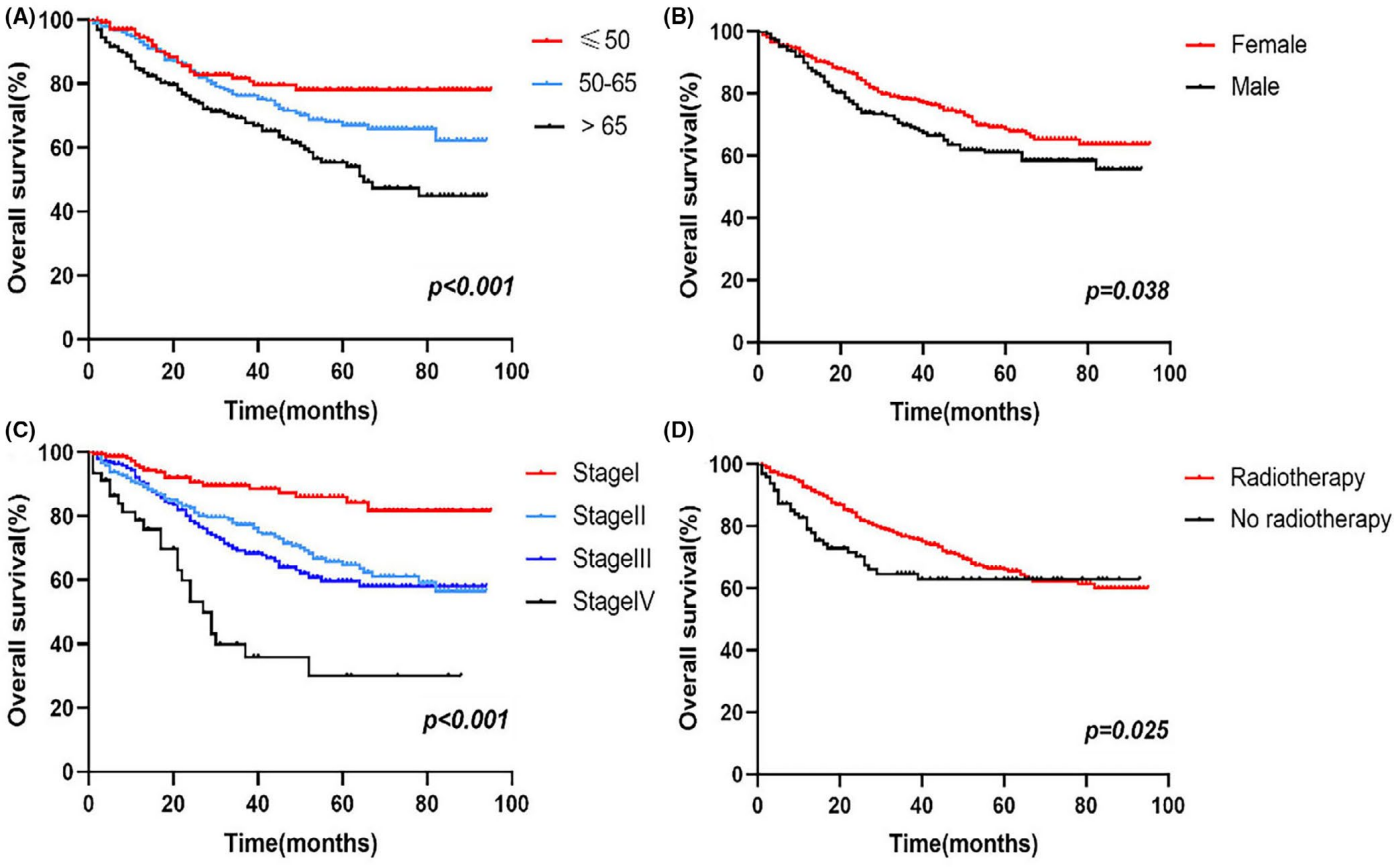
Kaplan–Meier survival curves for ASCC patients in the training group including age (A), sex (B), AJCC stage (C), and radiotherapy (D)

In addition, multivariate analysis revealed that age (50–65: HR 1.886, 95% CI 1.165–3.052; >65: HR 2.983, 95% CI 1.839–4.840; ≤50 as a reference), sex (female: HR 0.642, 95% CI 0.473–0.870; male as a reference), AJCC stage (II: HR 3.788, 95% CI 1.614–8.891; III: HR 2.046, 95% CI 0.661–6.335; IV: HR 4.786, 95% CI 1.505–15.226; I as a reference), and radiotherapy (radiotherapy: HR 0.559, 95% CI 0.365–0.857; no radiotherapy as a reference) were all correlated with OS (*p *< 0.05). Survival analysis showed that compared to cancer patients aged ≤50 years, patients aged 50–65 years had a worse prognosis, followed by those aged >65 years, and the differences were statistically significant (Figure 2A); The prognosis of male patients with ASCC was worse than those of female patients (Figure 2B). In addition, the more advanced the patients’ AJCC stage was, the worse the prognosis was (Figure 2C). Patients who received radiotherapy had a better prognosis (Figure 2D).

### 3.3 Construction and validation of the nomogram

**FIGURE 3:**
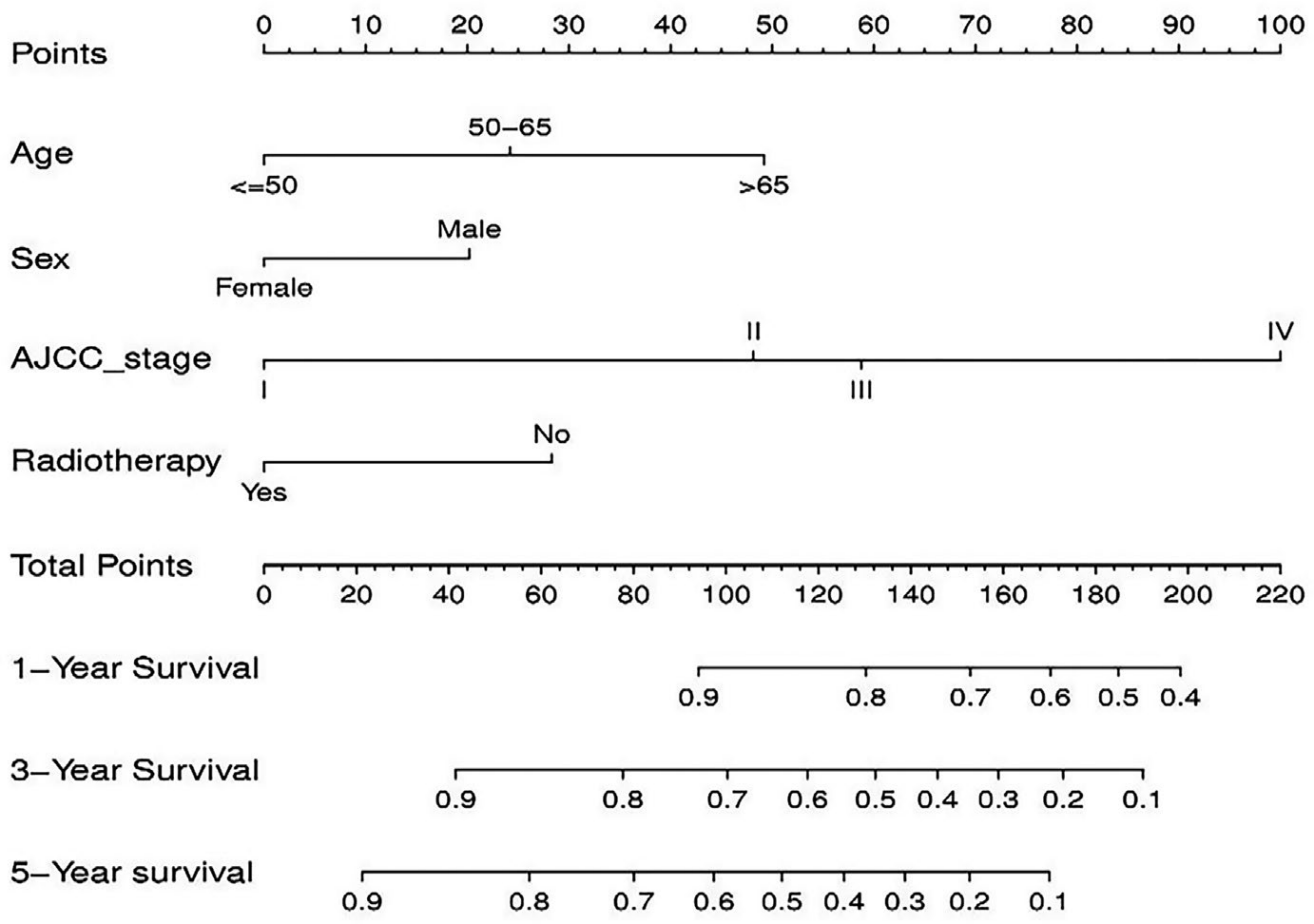
Nomogram for predicting 1‐, 3‐, and 5‐year survival rate of ASCC patients

On basis of the prognostic factors of OS derived from the Cox proportional hazard regression analyses in the training group, a nomogram was developed for predicting ASCC patients’ survival probability in 1‐, 3‐, and 5‐years (Figure 3). As shown in the nomogram, AJCC stage contributed the greatest significant influence on patients’ survival outcome, followed by age, radiotherapy, and sex. To obtain access to a patient's survival probability by nomogram, we need to identify the exact score of the four factors in ASCC patients (Table 3), totalize the scores and find the corresponding 1‐, 3‐, and 5‐year survival probabilities based on the total score.

**TABLE 3:** The scores of each variable

Variables	Nomogram
Age	
≤50	0
50–65	24.2
>65	49.2
Sex	
Female	0
Male	20.2
AJCC stage	
I	0
II	48.1
III	58.8
IV	100
Radiotherapy	
Yes	0
No	28.3

**FIGURE 4:**
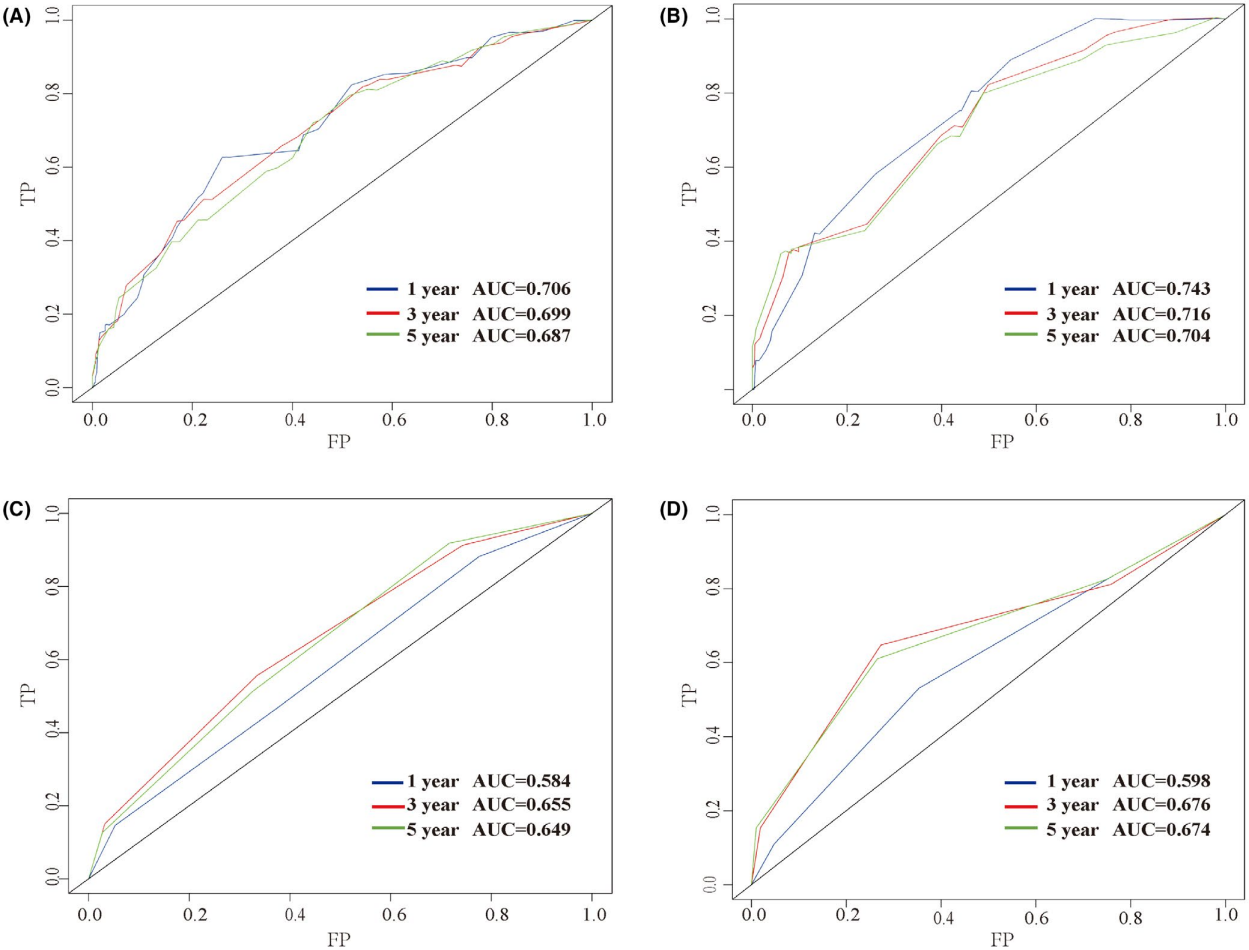
ROC curves for predicting the 1‐, 3‐, and 5‐year OS in the training group and in the validation group by nomogram (A, B) and AJCC stage (C, D)

**FIGURE 5:**
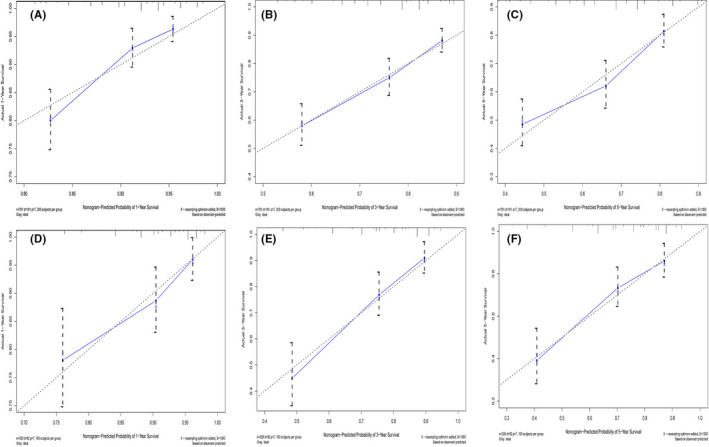
The calibration plot assessing the consistency between predicted and observed survival rates in 1‐, 3‐, and 5‐ years in the training group (A–C) and the validation group (D–F). The X‐axis stands for the survival predicted by the nomogram; Y‐axis represents actual survival. The graph along the 45‐degree line manifests the ideal calibration model, in which the predicted rate is in concert with the actual probability

Furthermore, the C‐index, AUC, and calibration curves were employed to test our prognostic model. Using random sampling of internal and external cohorts to verify the prediction performance of the model, the C‐index of the nomogram was 0.684 (95% CI, 0.643–0.725) and 0.730 (95% CI, 0.677–0.783) in the training and validation groups, respectively, exhibiting a good discrimination capability of the model to predict the survival probability of ASCC patients. The 1‐, 3‐, and 5‐year AUC values were 0.706, 0.699, and 0.687 in the training cohort (Figure 4A) and 0.743, 0.716, and 0.704 in the validation cohort, respectively (Figure 4B). In addition, the calibration plots displayed favorable consistency between the observed 1‐, 3‐, and 5‐year survival rates and the predicted rate, regardless of the training group (Figure 5A–C) or the validation group (Figure 5D–F), indicating that the nomogram was reliable.

### 3.4 Comparison of the nomogram and AJCC staging system

**FIGURE 6:**
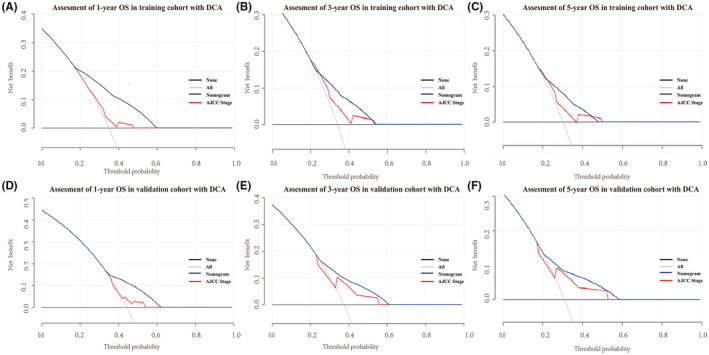
DCA to evaluate the 1‐,3‐, and 5‐year OS in the training group (A–C) and the validation group (D–F) by nomogram and AJCC stage. The horizontal line parallel to the axis stands for the total death of no patients, while the solid gray line shows the total death of all patients under a certain threshold rate

To further verify the superiority of our model, we compared our prognostic nomogram with the AJCC staging system. We found that the C‐index of the AJCC stage for OS was 0.610 (95% CI: 0.569–0.651) and 0.659 (95% CI: 0.598–0.720) in the training and validation groups, respectively, lower than that in the nomogram. We further calculated the AUC values of 1‐, 3‐, and 5‐year to evaluate the model's discrimination. The results showed that the nomogram was superior to the AJCC stage in the training and validation groups (Figure 4). The DCA also showed that the net return of our prognostic model exceeded that in the AJCC stage for a wide range of threshold rates (Figure 6). These results suggest that our nomogram can better predict the survival probability of ASCC patients.

### 3.5 Risk stratification via the nomogram

**FIGURE 7:**
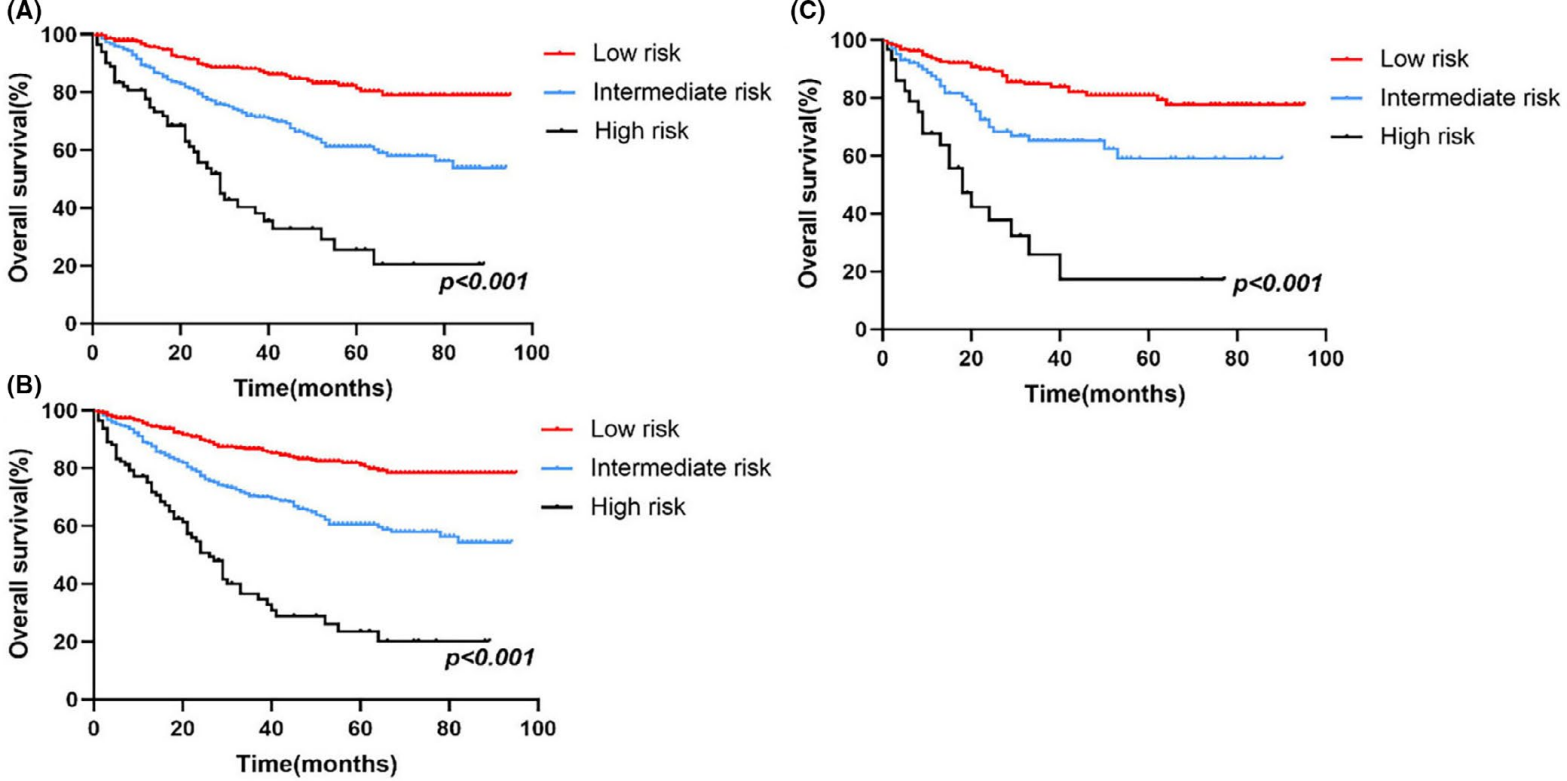
Kaplan–Meier curves of the low‐, medium‐, and high‐risk groups in the training cohort (A), the validation group (B), and all patient's group (C)

Furthermore, in order to improve the management of patients with ASCC, a risk stratification model was developed to divide patients into three groups: low‐risk (total scores <73), medium‐risk (73 ≤total scores <121), and high‐risk (total scores ≥121) groups, using the X‐tile software to determine the optimal cutoff values based on the total scores of every patient (Figure S2). Kaplan–Meier analysis was conducted in the training, validation and all groups, and an obvious difference existed in the OS (Figure 7), indicating the practical utility of our prognostic nomogram in risk stratification for patients with ASCC.

## 4 DISCUSSION

In this study, 1087 ASCC patients were enrolled from the SEER database and four prognostic considerations were identified from 13 variables to develop a nomogram to better predict patient survival probability. The C‐indexes, ROC curves, and calibration curves confirmed the reliability and discrimination of the model and the DCA proved its clinical utility. Furthermore, a risk stratification model was developed to classify patients into low‐risk, medium‐risk, and high‐risk groups, which may help clinicians to improve patient management. It is the first large retrospective study to establish a nomogram to predict the prognosis of ASCC patients and these significant factors can be accessible to better predict the survival outcome of each patient and aid clinicians in making appropriate therapy decisions.

In our study, age was identified as a significant independent risk factor associated with OS in patients with ASCC. The incidence and mortality of ASCC is increasing, with an approximate 5% increase in SCCA‐based mortality in patients aged 60–69 years.^2^ Compared to younger patients, elderly patients are less likely to receive standard‐of‐care chemoradiotherapy. In addition, considering the adverse effects, when chemoradiotherapy was delivered, the elderly preferred to receive concurrent single‐agent rather than multiagent chemotherapy, which resulted in a worse survival outcome.^10^, ^20^, ^21^

In addition, compared to female patients, male patients had a worse survival outcome, which is consistent with the results of previous studies.^11^, ^14^, ^22^, ^23^ Some researchers have argued that women are more active in their health care, receive more cancer screening, and are more likely to be diagnosed at earlier stages of the disease. There are well‐known risk factors for HPV and HIV infection, prompting the development of ASCC.^5^, ^24^, ^25^ Shiels et al.^26^reported that HIV infection was the main reason for the increased incidence of male patients with ASCC. Men who were engaged in anal intercourse had an increased risk of ASCC.^27^, ^28^, ^29^, ^30^, ^31^, ^32^, ^33^ In addition, single men had a higher incidence of anal cancer than married men.^34^, ^35^ In the entire cohort, there were 402 male patients, among whom 272 were single and 130 were married; for the 685 female ASCC patients, 382 and 303 were single and married, respectively. Over 60% of male patients are single, which may contribute to a higher risk for ASCC. For male patients with ASCC, a high risk of HIV infection may lead to a poor prognosis in this population.

The literatures have had inconsistent results on the presence of marital and racial differences on survival of ASCC patients. Wu et al.^36^ analyzed 2111 localized anal carcinoma patients who received definitive chemoradiation between 2004 and 2012 and found the married and the white positively impacted survival. Marriage may be a proxy for social support, which is essential among cancer survivors because of the great psychosocial burden.^37^ In addition, it is controversial whether black race was independently associated with worse survival.^38^, ^39^ In our study, univariate analysis showed that the married and the white could reduce patients’ death risk, but further multivariate analysis showed that they were not independent prognostic factors. These results’ differences could be owing to an interplay of structural, cultural, and social issues associated healthcare.

According to the prognostic nomogram model, AJCC stage had the largest influence on the survival prediction of ASCC patients. As shown in the comparative analysis of the prognostic model and the AJCC staging system, it can be seen that the AJCC stage plays a certain role in the survival prediction of ASCC patients. It can also be said that our predictive model is a further improvement of the AJCC staging system based on patients’ demographic information and clinicopathological features.

In terms of the treatment, the current standard‐of‐care for localized ASCC is concurrent chemoradiotherapy (CRT). In the 1970s, Norman Nigro promoted organ conservation therapy and found that the clinical complete response rate to CRT was 86%.^40^, ^41^ Subsequently, CRT was identified as the standard‐of‐care for localized ASCC through two key trials: the Anal Cancer Trial (ACT I) of the UK Coordinating Council for Cancer Research (UKCCCR) and the European Organization for Research and Treatment of Cancer (EORTC).^6^, ^22^, ^42^ However, the optimal management of patients with stage I ASCC is controversial. Some studies have indicated that the combination with chemotherapy does not correlate with inferior OS.^19^, ^43^, ^44^, ^45^ In addition, for patients with well‐differentiated T1, N0, M0, or smaller T2 perianal tumors, local surgery with margins of 1 cm may be an effective treatment strategy.^46^ However, CRT cannot be ignored in patients who have received local resection. ASCC often metastasizes to the lung, liver, and extrapelvic lymph nodes although patients are rarely diagnosed with stage IV disease. Chemotherapy is advocated,^5^ but radiation for metastatic sites can also be regarded as part of combination therapy.^46^ In addition, immunotherapy may also provide survival benefits to patients who fail chemotherapy.^47^, ^48^ In our study, most patients were diagnosed as stage I‐III, which provides the opportunity for radiotherapy and chemotherapy for them and offer a relatively better survival outcome. Moreover, the majority of patients undergoing surgery also received radiochemotherapy. Univariate analysis showed that surgery and radiotherapy can reduce the patients’ death risk, and chemotherapy also seemed to improve patients’ survival outcomes, but the difference was not statistically significant. Further multivariate analysis showed that radiotherapy was an independent prognostic factor. Chemotherapy is an important treatment option for ASCC, but its actual efficacy may be underestimated in our study. In SEER database, chemotherapy is coded as “yes” or “no/unknown,” but the specific protocol and cycle cannot be obtained. Therefore, our results should be interpreted cautiously.

Nowadays, the trend is to treat rectal squamous cell carcinoma (RSCC) by analogy to ASCC.^49^ Because of the same histotype and close localization, we wonder if there are differences in prognostic factors between ASCC and RSCC. Diao et al.^50^ enrolled 806 RSCC patients and constructed a nomogram to predict 3‐ and 5‐years OS. However, compared with our study, different prognostic factors in demographic characteristics, AJCC stage and treatment options, were identified, which may be caused by the differences in pathogenesis,^51^ staging,^52^, ^53^ and treatment options^49^, ^54^ between ASCC and RSCC. Due to the rarity of RSCC, much is unknown about this cancer, such as the role of HPV and HIV in RSCC,^55^, ^56^ the molecular profile or the most effective treatments. And more studies are needed to address these questions.

There are some limitations in our study. First, this is a retrospective study, and there is a lack of data collection, including virus infection status, related molecular factors, and detailed treatment information, which may improve the accuracy of the model. Second, there may be selection bias due to the exclusion of patients with missing data. Finally, because of the rarity of the ASCC patients, we cannot externally validate the nomogram from patients in our hospital, and the prognostic model may be more applicable to patients in the United States.

## 5 CONCLUSION

In conclusion, a nomogram for patients with ASCC was constructed on the basis of four significant prognostic factors determined by univariate and multivariate Cox analysis. It enables clinicians to better anticipate the 1‐, 3‐, and 5‐year survival probabilities, and plays a role in risk stratification and treatment decision making for patients with ASCC.

## CONFLICT OF INTERESTS

The authors declare that they have no conflict of interests.

## AUTHOR CONTRIBUTIONS

Ningning Yang: Data acquisition, methodology, and writing original draft. Lu Xu: Methodology and writing original draft. Qingqing Wang: Methodology and revising the manuscript. Fengxia Chen: Writing assistance. Yunfeng Zhou: Conceptualization and project administration. All authors have read and approved the manuscript.

## ETHICAL APPROVAL STATEMENT

These public data are open access and do not need Institutional Review Board approval.

## Supporting information

Fig S1

Fig S2

## Data Availability

The SEER database is publicly available. The datasets used and/or analyzed during the current study are available from the corresponding author upon reasonable request.
