# Supplementary figures and images for: Construction and validation of a prognostic nomogram for anal squamous cell carcinoma
Source: Cancer Med. 2021 Dec 1;11(2):392–405. doi: 10.1002/cam4.4458 (PMC8729044; doi:10.1002/cam4.4458)

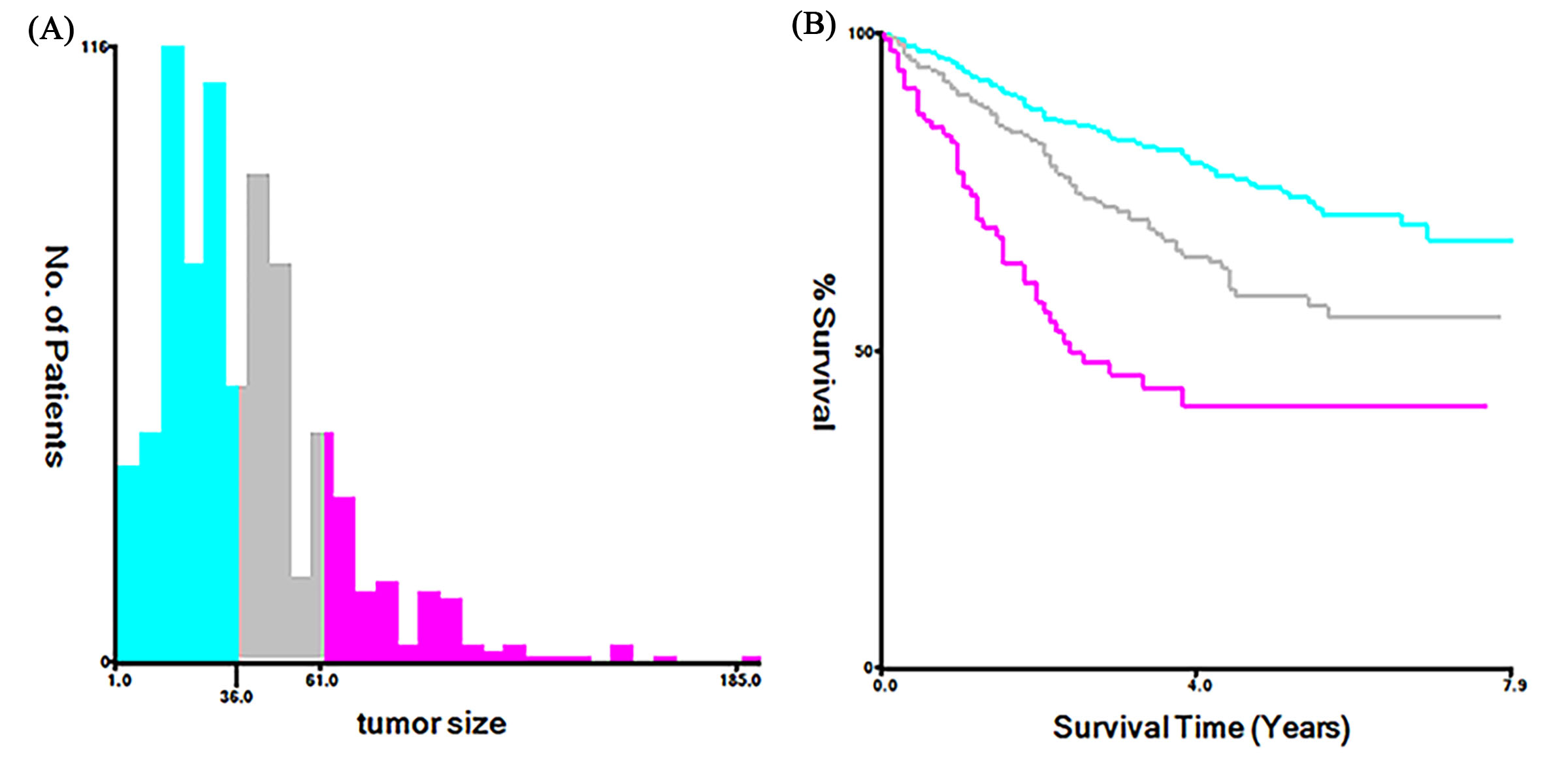

Supplement: Supplementary file 1 — Fig S1 [file CAM4-11-392-s001.jpg]

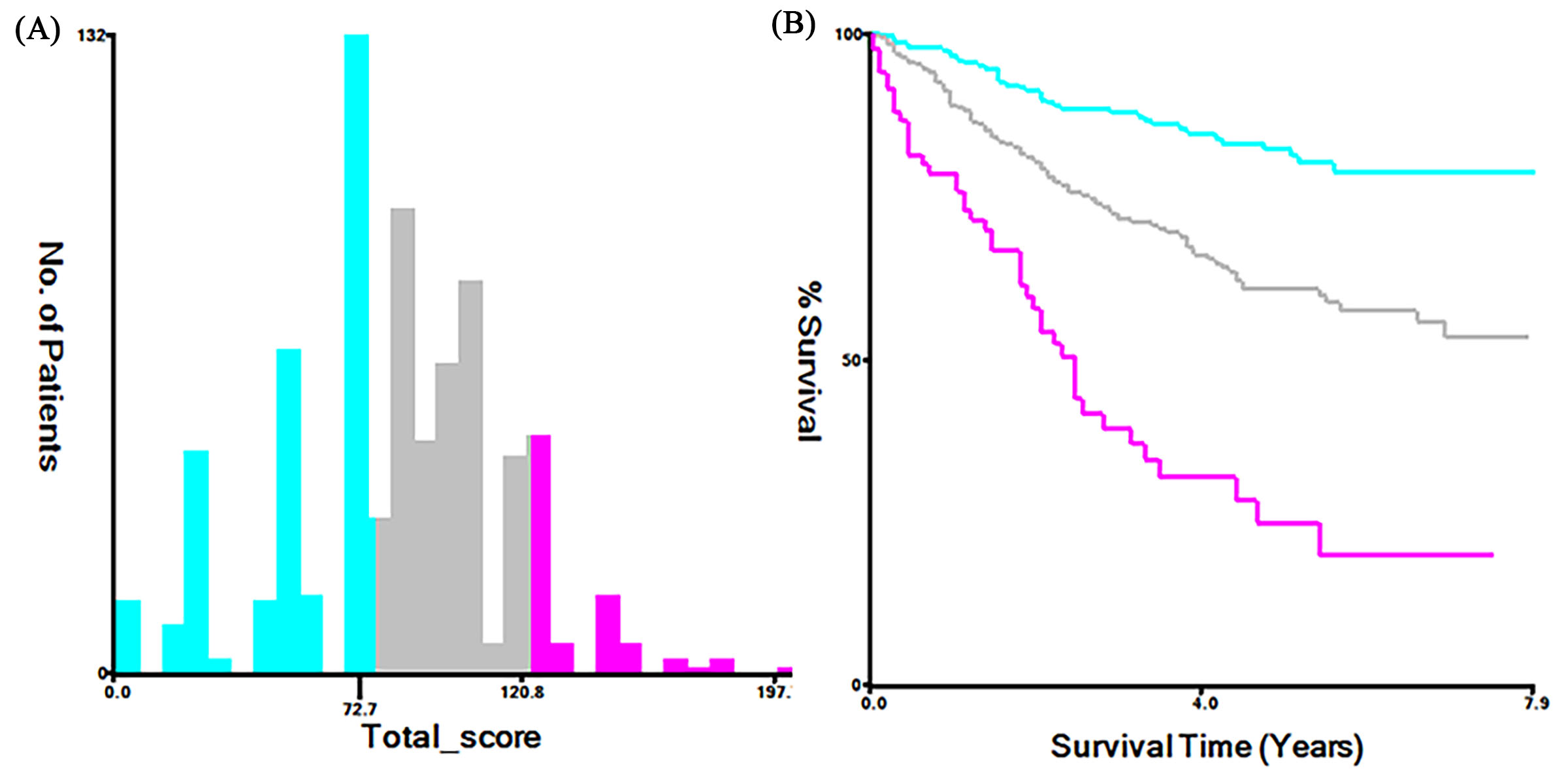

Supplement: Supplementary file 2 — Fig S2 [file CAM4-11-392-s002.jpg]
